# Forecasting extreme atmospheric events with a recurrence-interval-analysis-based autoregressive conditional duration model

**DOI:** 10.1038/s41598-018-34584-4

**Published:** 2018-11-02

**Authors:** Yue-Hua Dai, Zhi-Qiang Jiang, Wei-Xing Zhou

**Affiliations:** 1 School of Business, East China University of Science and Technology, Shanghai, 200237 China; 2 Research Center for Econophysics, East China University of Science and Technology, Shanghai, 200237 China; 3 Department of Mathematics, East China University of Science and Technology, Shanghai, 200237 China

## Abstract

With most city dwellers in China subjected to air pollution, forecasting extreme air pollution spells is of paramount significance in both scheduling outdoor activities and ameliorating air pollution. In this paper, we integrate the autoregressive conditional duration model (ACD) with the recurrence interval analysis (RIA) and also extend the ACD model to a spatially autoregressive conditional duration (SACD) model by adding a spatially reviewed term to quantitatively explain and predict extreme air pollution recurrence intervals. Using the hourly data of six pollutants and the air quality index (AQI) during 2013–2016 collected from 12 national air quality monitoring stations in Beijing as our test samples, we attest that the spatially reviewed recurrence intervals have some general explanatory power over the recurrence intervals in the neighbouring air quality monitoring stations. We also conduct a one-step forecast using the RIA-ACD(1,1) and RIA-SACD(1,1,1) models and find that 90% of the predicted recurrence intervals are smaller than 72 hours, which justifies the predictive power of the proposed models. When applied to more time lags and neighbouring stations, the models are found to yield results that are consistent with reality, which evinces the feasibility of predicting extreme air pollution events through a recurrence-interval-analysis-based autoregressive conditional duration model. Moreover, the addition of a spatial term has proved effective in enhancing the predictive power.

## Introduction

Regardless of the air quality monitoring stations’ commitment to present the latest air quality reports and exhaustive air quality information, Chinese residents are tending to suffer a longer stretch of stifling air pollution periods^1^. As such, a high premium has been put on explaining and forecasting the occurrence of extreme atmospheric events due to their influence on people’s daily life and health^2,3^. Unlike other extreme events, the occurrence of extreme air pollution events is rarely studied for the following reasons^2^. First, the gaps between extreme value theory and its applications in atmospheric time series still exist. Second, the air monitoring stations have begun to offer high-frequency data only in recent years. In this paper, to model the recurrence intervals of extreme air quality events, we resort to the method that is widely used in modeling the financial market risk^4–6^.

Research efforts to evaluate and predict the occurrence of extreme air pollution have been made from various perspectives^2,3,7^. Wang *et al*. established an early-warning system using a hybrid forecasting model based on some data processing methods (such as support vector machine and fuzzy set theory)^2,3^. Whereas their quest for optimal distributions to model the air pollution time series is partially similar to ours, differences do exist in the models to deal with the distributions. We gravitate towards the recurrence frequency of the extreme events and their spatiotemporal properties yet they followed with interest the distributions and model selections in evaluating the time series. Niu *et al*. proposed the ensemble empirical mode decomposition and least square support vector machine (EEMD-LSSVM) method based on phase space reconstruction (PSR) to analyze the pollution time series^7^. Their results boast a higher predictive accuracy when applied in Lanzhou and Guangzhou. Other relevant studies adopt multifractal analysis to check the long-term or short-term structure and self-organized properties of recurrence intervals. More importantly, with both numerical and model-based analytical approaches, prediction making and variants of Value-at-Risk estimates have been discussed intensively in recent literature based on the statistics of return intervals, conditional intervals and event expectation times^8–11^. However, we steer the wheel to another direction which focuses on the ARMA structure and spatial explanatory power. It’s worth to mention that Beck and Cohen put forward a two-compound superstatistical model to model multiple fragments characterized by the exponential inter-session time distribution, which has been widely used in complex systems for risk estimation^11–15^.

In this paper, we integrate the recurrence interval analysis with the autoregressive conditional duration model to measure the recurrence statistics of extreme air pollution events, which accords with Herrera and Schipp’s methodology to predict the value at risk of stock market index^16^. In addition, peaks over threshold (POT) method is also employed to generate POT series and the autoregressive conditional duration (ACD) model is similarly applied to analyze the generated series. In this regard, our paper follows Herrera and Schipp’s framework and tries to apply the ACD model into the recurrence intervals analysis. However, given the spatial characteristics of the air pollution time series, we incorporate the spatial term into the ACD model and attempt to unveil the spatial predictive power of neighbouring stations. In this study, we first analyze the basic statistics of recurrence interval series under different thresholds. We also propose the spatially reviewed recurrence intervals by adding another monitoring station as a benchmark to fully incorporate spatial reference information. Using the spatially reviewed recurrence interval as an exogenous variable in the conditional duration model, this paper extends the ACD model to a spatial ACD model. With the help of maximum likelihood estimation (MLE), we find that some coefficients of the spatial term are significant at 1% level, which indicates a strong predictive power of the spatially reviewed recurrence intervals. On top of that, this paper also goes from the simplest scenario to more temporal lags and spatially neighbouring stations to evaluate how the predictive power changes with time and distance. Finally, in this paper, we conduct a one-step forward forecast using both the classic ACD model and the spatial ACD model. Statistics show that 90% of the predicted recurrence intervals deviate by less than 72 hours from the actual value, which manifests the predictive power of the proposed model. Moreover, there is evidence that the spatial ACD model is slightly better than the ACD model due to the use of spatial information in predicting extreme pollution events.

## Results

### Recurrence intervals

A recurrence interval analysis pertains to the time intervals between two consecutive extreme events^17,18^. It has been widely studied both in nature science^18–21^ and social science^4,22,23^. For a given pollutant and threshold, long intervals indicate its inactivity which may then signify a period of weak industrial activity and gentle meteorological fluctuations. Conversely, increased industrial activities and unstable climatic conditions may give rise to excess concentration of the pollutant and result in shorter intervals. The dynamic behavior of the durations thus contains valuable information about both the human activities and meteorological changes.

The recurrence interval *d*_*q*_ is defined as the waiting time between two consecutive events in which the normalized concentration *x* exceeds the threshold *q*.

1 $${d}_{q}=\,{\rm{\min }}\{t-t^{\prime} :x(t) > q,\,x(t^{\prime} ) > q,\,t > t^{\prime} \}$$

**Figure 1:**
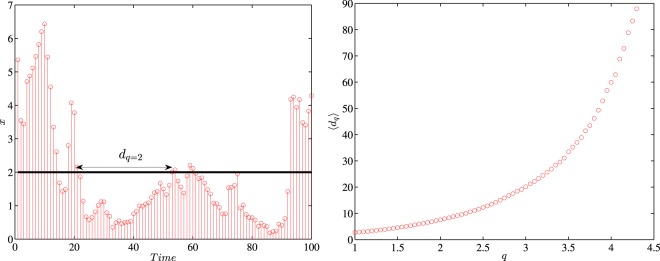
An illustrative example of the recurrence interval *d*_*q*_ (left panel) and the relationship between *q* and 〈*d*_*q*_〉 (right panel). The selected *x* is diurnally adjusted PM_2.5_ time series at station 1.

In this paper, the selected *q* values range from 2.0 to 4.0 with an increment of 0.5. Generally speaking, when *q* = 2.0, the air condition is categorized as mild pollution, whereas when *q* = 4.0, it is labelled as serious pollution. Specifically, judging from Eq. (2) and Fig. 1(b), it’s apparent that *q*s in this bulk account for around 10% to 1% extreme air pollution conditions^5^

2 $$\frac{1}{\langle {d}_{q}\rangle }={\int }_{q}^{+\infty }\,p(x)dx$$

**Figure 2:**
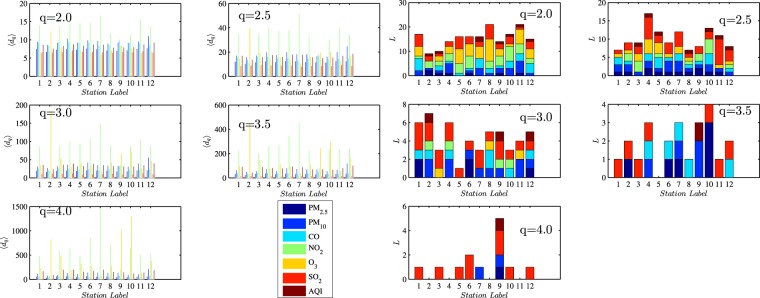
Average recurrence intervals (left panel) and AR(L) models (right panel) under five different *q*s. In the right panel, under each threshold *q*, we first obtain the recurrence interval series *d*_*q*_ and then model *d*_*q*_ using the autoregressive model. The maximal significant (95% confidence interval) lag is denoted as *L*. For one station, the *L*s of the six pollutants in question and AQI’s *d*_*q*_s are stacked in bars with distinct colors.

When it comes to different pollutants under each threshold, Fig. 2 presents how the average recurrence interval 〈*d*_*q*_〉 varies and how temporally persistent the recurrence interval series are. Generally, the recurrence intervals of NO_2_ and O_3_ are substantially higher than those of other pollutants, and this trend is especially true of higher *q*s. Thereby it can be concluded that fine particulate matters are the main pollutants of air pollution and this is congruent with the previous findings^24,25^. Apart from these two pollutants, pollution stemming from four other pollutants happens about every 10 hours on average and this tendency may be attributed to the diurnally cyclical patterns of air pollutants^26^. As *q* rises from 2 to 4, the recurrence intervals for these four pollutants are almost doubled. However, NO_2_ and O_3_ show a different picture, with the average recurrence intervals around 15 hours when *q* = 2 and rocketing to 1000 hours when *q* = 4, which suggests that these two pollutants are marginal in air pollution. The average recurrence intervals are also scrutinized by the monitoring stations. Figure 2(a) shows that all these stations share similar recurrence intervals on the aforementioned four pollutants and differ in the average recurrence intervals of NO_2_ and O_3_. In station 2, the average recurrence intervals for O_3_ outweigh all other pollutants, and this is true for stations 9 and 10 when *q* rises. Finally, AQI, as a comprehensive air quality index, overtops *q* = 2 every 10 hours, and the interval is doubled when *q* increases by 0.5. This trend strong evidence that air pollution deserves to be put on the list of priorities^1^.

On the temporal side, autoregressive model AR(L) is employed to fit *d*_*q*_s for each adjusted time series to identify the autocorrelation structure of recurrence intervals. The first *L* significant (under 95% confidence interval) lags for *d*_*q*_s are stacked in Fig. 2(b). The general trend shows that as the threshold increases, the autocorrelation is weakening across both monitoring stations and pollutant categories. When *q* = 4, SO_2_ becomes the only pollutant whose recurrence intervals still autocorrelate. Two possible reasons may account for this. Firstly, the threshold is selected mainly based on normalized AQI and may be somewhat lower for normalized SO_2_ series, yet this alternative is ruled out after a scrutiny of the original data. Secondly, it’s more likely that the SO_2_’s autocorrelation structure is persistent compared with other time series^27,28^. As the threshold *q* climbs up to 3, the autocorrelations of most air pollutants’ recurrence intervals are diminishing, which inspires our exploration of the correlation structure from the spatial side.

### Scaling behaviour of the recurrence intervals

**Figure 3:**
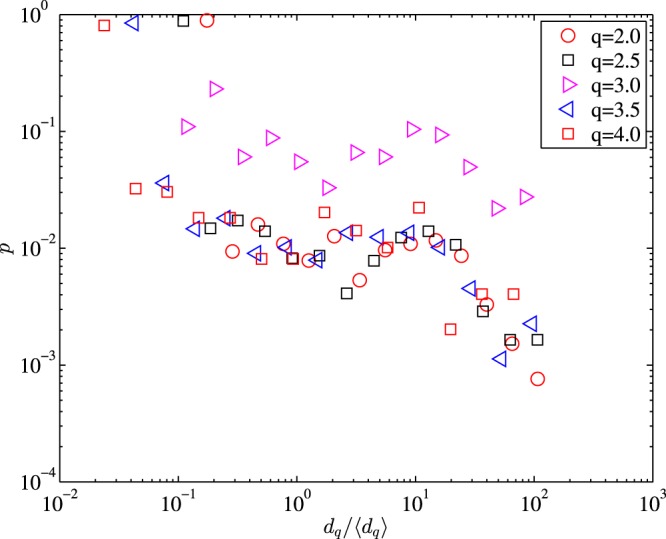
The probability of *d*_*q*_/〈*d*_*q*_〉 of the PM_2.5_ series in station one.

As many other natural phenomena^29–31^, in this section, we try to check whether there exist scaling properties in the original time series and recurrence intervals series of the air pollutants. In Fig. 3, it’s straightforward that most of the recurrence intervals occur between 0.01〈*d*_*q*_〉 and 0.1〈*d*_*q*_〉 and much smaller than 〈*d*_*q*_〉. Longer inter-event intervals are less likely to occur. However, due to long-term correlation of the concentration time series of PM_2.5_^26^, the intervals series show only several unique numbers, making it hard to capture the term structure of the recurrence intervals series of the air pollutants. When it comes to different thresholds, the scaling behavior still shows no traceable patterns and almost 99% of the recurrence intervals are 1 hour, indicating that the air pollution is followed by one another and recur in clusters.

**Figure 4:**
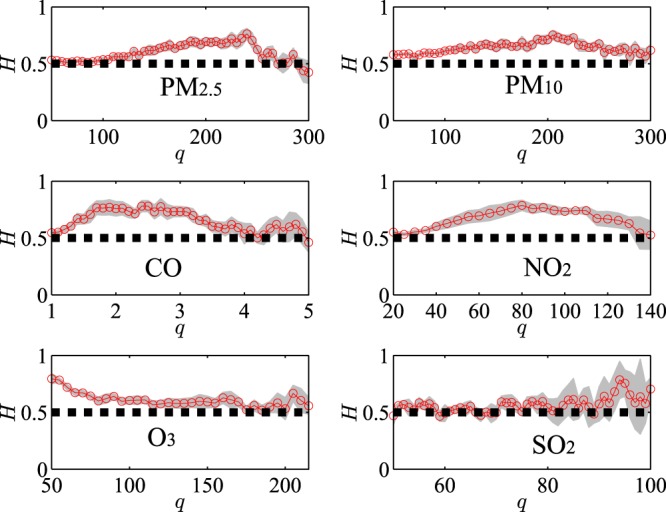
The Hurst exponent and the threshold for the six pollutants at Station 1.

When checked the long-term correlation of the air pollutants at different locations, we do believe the finite sample size and data quantization effects influence the results^32^. Factually, sample size and data quantization affect the results through several channels. First, as *q* is lifted, less recurrence data will be generated, and the long-term correlation structure is influenced. Especially, as *q* = 4, the precision of the long-term correlation is certainly reduced, the choice between long-term correlation and short-term correlation is thus affected. Secondly, as *q* is rising, less data will lead more bias and convergence problems when calculating the Autoregressive Conditional Duration Model. To consolidate the influence of the sample size effect, we use the original data to check the long-term correlation, and as evinced in Fig. 4, when the threshold *q* is very high, the sample size is shortened, and the Hurst exponent will be indistinguishable from 0.5 which signals that it is the very hard to identify the term structure as the sample size is limited.

### Spatially reviewed recurrence intervals

**Figure 5:**
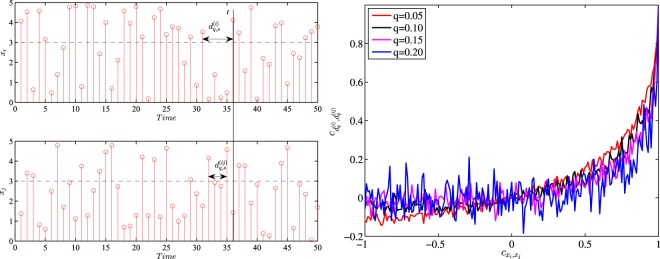
An illustrative example of ${d}_{q,s}^{(i)}$ and its spatially reviewed recurrence intervals ${d}_{q,s}^{(ij)}$, *q* = 3.0 in this example (left panel) and simulated relationship between the original time series’ correlations and recurrence interval correlations under different thresholds (right panel). In the right panel, first we simulate standard distribution time series pairs and then use the spatially reviewed recurrence intervals scheme to obtain the ${d}_{q}^{(i)}$ and ${d}_{q}^{(ij)}$ and calculate the correlation.

In this part, we define the recurrence intervals from the spatial perspective. For two locations *i* and *j*, we define *j*’s spatially reviewed recurrence intervals using *i*’s recurrence intervals as the time breakpoint: Whenever location *i*’s recurrence ends, the most recent recurrence interval at the same threshold *q* at location *j* is denoted as *j*’s spatially reviewed recurrence interval ${d}_{q}^{(ij)}$. In this definition, we introduce *i* as a benchmark. Whenever the extreme pollution spell terminates, people at location *i* will receive a message from location *j* about how long the most recent recurrence lasts in *j* under the same threshold *q*. As Fig. 5(a) illustrates, these two locations will have observed their last recurrence intervals under the same threshold *q*. A problem may arise when *t* is too small, and location *i* has observed an occurrence while location *j* has not. In this case, we, ad hoc, adopt the average interval at location *j* as its spatially reviewed recurrence interval. In this way we can generate two equal lengths of recurrence interval series ${d}_{q}^{(i)}$ and ${d}_{q}^{(ij)}$. It should be noted that ${d}_{q}^{(ij)}$ is not exactly the same as ${d}_{q}^{(j)}$ at location *j* without any spatially reference introduced if generated with the above method. However, they will remain in high “correlation” with the single time series’ recurrence intervals. Nonetheless this correlation is hard to measure because of the unequal lengths of the two series.

We simulate two independent standard distribution series *x*_*i*_ and *x*_*j*_ with correlation ${c}_{{x}_{i},{x}_{j}}$ varying from −1 to 1. Figure 5(b) shows *i*’s recurrence interval series ${d}_{q}^{(i)}$ and *j*’s spatially reviewed recurrence intervals ${d}_{q}^{(ij)}$ are of no significant correlation when ${c}_{{x}_{i},{x}_{j}}$ is lower than 0.5. However, when ${c}_{{x}_{i},{x}_{j}}$ is higher than 0.5, their recurrence intervals apparently becomes more correlated, which indicates that if the two original time series are of high correlations under the identical mean, variance and distribution conditions, the recurrence intervals of one series are more likely to be strongly and positively correlated with its neighbour’s spatially reviewed recurrence intervals. In other words, if location *i*’s concentration time series are in high correlation with *j*’s, the message about recurrence intervals at location *j* is conducive to explaining and predicting the recurrence intervals at *i*.

Excitingly, Table 1 panel A shows that all the minimum correlations of the 7 selected time series between the 12 stations are over 0.50, which indicates a strong likelihood that one station’s recurrence interval series over a threshold are also highly correlated with its neighbour’s spatially reviewed recurrence intervals. This euphoria of high spatial correlation structure in reviewed recurrence intervals fuels our interest in extending traditional time series models to more general spatiotemporal models. The inter-station distances as tabulated in panel B are all below 100 *Km*, which has twofold implications. First, once the inter-station distance is longer than 100 *Km*, it’s likely to introduce the variations of geographical and meteorological conditions to this model and reduce the explanatory and prediction powers of the neighbouring stations. Second, when observing the air conditions and the frequency of extreme pollution events, people are more apt to refer to the situation of an adjacent area instead of remote ones. To recapitulate, the aforementioned reasons are the main inspirations to define the recurrence intervals from the spatially reviewed angle.

**Table 1:** Minimum correlation matrix and distance matrix between stations.

	1	2	3	4	5	6	7	8	9	10	11	12
**Panel A: Minimum correlation matrix between stations**.												
1	1.00	0.60	0.91	0.89	0.89	0.92	0.85	0.73	0.64	0.68	0.87	0.83
2	0.60	1.00	0.59	0.59	0.59	0.61	0.61	0.63	0.74	0.80	0.58	0.63
3	0.91	0.59	1.00	0.85	0.93	0.92	0.82	0.71	0.60	0.67	0.90	0.79
4	0.89	0.59	0.85	1.00	0.86	0.84	0.79	0.68	0.59	0.68	0.81	0.78
5	0.89	0.59	0.93	0.86	1.00	0.90	0.82	0.74	0.61	0.68	0.88	0.79
6	0.92	0.61	0.92	0.84	0.90	1.00	0.86	0.72	0.63	0.70	0.88	0.81
7	0.85	0.61	0.82	0.79	0.82	0.86	1.00	0.73	0.66	0.67	0.81	0.83
8	0.73	0.63	0.71	0.68	0.74	0.72	0.73	1.00	0.72	0.69	0.72	0.72
9	0.64	0.74	0.60	0.59	0.61	0.63	0.66	0.72	1.00	0.73	0.58	0.66
10	0.68	0.80	0.67	0.68	0.68	0.70	0.67	0.69	0.73	1.00	0.65	0.68
11	0.87	0.58	0.90	0.81	0.88	0.88	0.81	0.72	0.58	0.65	1.00	0.77
12	0.83	0.63	0.79	0.78	0.79	0.81	0.83	0.72	0.66	0.68	0.77	1.00
**Panel B: Distance matrix between stations. (** ***Km*** **)**												
1	0.00	49.53	11.08	5.86	14.76	8.38	14.69	43.08	63.15	38.27	15.51	13.79
2	49.53	0.00	43.45	51.05	43.49	41.58	34.86	49.35	41.92	11.36	37.42	40.17
3	11.08	43.45	0.00	8.64	3.96	6.32	11.13	32.37	52.26	32.13	6.11	18.03
4	5.86	51.05	8.64	0.00	11.30	9.80	16.67	38.62	60.46	39.68	14.49	18.80
5	14.76	43.49	3.96	11.30	0.00	10.08	13.68	28.45	49.16	32.35	6.63	21.70
6	8.38	41.58	6.32	9.80	10.08	0.00	6.89	37.90	55.66	30.24	7.80	11.71
7	14.69	34.86	11.13	16.67	13.68	6.89	0.00	38.32	52.58	23.59	7.91	10.58
8	43.08	49.35	32.37	38.62	28.45	37.90	38.32	0.00	28.54	42.04	30.90	48.52
9	63.15	41.92	52.26	60.46	49.16	55.66	52.58	28.54	0.00	41.49	47.89	62.88
10	38.27	11.36	32.13	39.68	32.35	30.24	23.59	42.04	41.49	0.00	26.15	29.74
11	15.51	37.42	6.11	14.49	6.63	7.80	7.91	30.90	47.89	26.15	0.00	17.63
12	13.79	40.17	18.03	18.80	21.70	11.71	10.58	48.52	62.88	29.74	17.63	0.00

The minimum correlation matrix is the minimum value of seven correlations between two stations.

### Primary results of RIA-ACD(1,1) and RIA-SACD(1,1,1)

Starting from the simplest scenario, we evaluate the RIA-ACD(1,1) and the RIA-SACD(1,1,1) models on the 84 selected time series respectively. To fully incorporate the recurrence intervals’ distribution information, the results are displayed from both exponential distribution and Weibull distribution of *ε*. Some scholars opted for power-law distribution with an exponential cutoff and q-exponential distribution to shelter the recurrence intervals’ heavy tails in stock market^4,5^. Simiu and Heckert showed that the recurrence intervals of wind data are better fitted by General Pareto distribution in the tails^33^. When checking the distributions of recurrence intervals of air pollutants, we find General Pareto distribution, with more parameters, to be more adaptive. However, involving more parameters in the distributions of standardized innovations surely means more complexity and instability of the estimation process though the results may be more accurate. To strike a balance between generality and complexity, we select the above two distributions. As noted before, in order to make the distributions meet the trend of recurrence intervals, we confine the shape parameter *k* in the Weibull distribution within [0, 1]. As the Weibull distribution possesses one more parameter than the exponential distribution, without loss of the precision and generality, this work reports the results for the Weibull distribution after presenting the primary results.

Tables 2 and 3 tabulate partial results of all the time series. While Table 2 mainly focuses on whether RIA-ACD model is valid in evaluating the expected recurrence intervals^16^, Table 3 probes whether incorporating spatial information in the above models improves the predictive power. In Table 2, the two distributions produce similar results for the estimated parameters *ω*_*q*_, *α*_*q*,1_, *β*_*q*,1_ and *Q*(10). Although some estimations may vary in significance, the estimated magnitudes of results are quite close under both exponential distribution and Weibull distribution. The diagnostic statistics *Q*(10) s are never significant in the first 10 lags for the normalized innovations, which indicates that the RIA-ACD model is successful in capturing the persistent features of the recurrence intervals in the time dimension^34^. Both exponential and Weibull distribution are successful in identifying the recurrence intervals’ probability distributions. In Table 2, as the value of *q* varies from 2.0 to 4.0, *β*_*q*,1_ is more likely to stay significant than *α*_*q*,1_, indicating that the conditional expected recurrence intervals’ autocorrelation structure is more stable. In other words, in predicting the recurrence intervals of more severe atmospheric pollution, the impact from last conditional expected interval *ψ*_*q*,*s*−1_ is more helpful than last realized interval *d*_*q*,*s*−1_. Another notable finding is that as *q* increases, the shape parameter *k* of Weibull distribution decreases, the implication of which is quite straightforward: The most extreme polluted days are rare and moderately polluted days are found within longer periods and the length of recurrence interval series is thus shortened, making the distribution flatter. In this sense, the declining *k* is quite consistent with the intuition^35^. But caution should be used to interpret the empirical meanings of the coefficients *α*_*q*,1_ and *β*_*q*,1_. As noted before, *α*_*q*,1_ mainly measures how significantly last realized recurrence interval influences this expected recurrence interval, and *β*_*q*,1_ evaluates how significantly last conditional expected recurrence interval influences this expected recurrence interval. For example, when *q* = 3, with the recurrence interval series of PM_10_ in station 2 under the exponential distribution, *α*_*q*,1_ = 0.26, which means that once last recurrence interval increases by 1 hour, it’s more likely that the expected interval increases by 0.26 hours on average. *β*_*q*,1_ = 0.46 shows that once last conditional expected interval increases by 1 hour, the expected interval increases by 0.46 hours on average. Under the stationary conditions, the future expected recurrence interval is *ω*_*q*_/(1−*α*_*q*,1_−*β*_*q*,1_) = 1.11〈*d*_*q*_〉.

**Figure 6:**
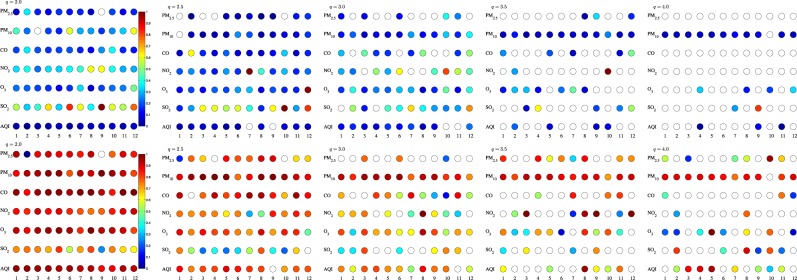
*The coefficients α*_*q*,1_ and *β*_*q*,1_ of each time series are estimated from the RIA-ACD(1,1) model under Weibull distribution. The magnitudes of the parameters are represented by different colors filled in each circle, and the circle with no color filled in signifies that the estimated parameter is not significant at 95% level. The top row is the *α*_*q*,1_ and the bottom row is the *β*_*q*,1_. Presented from the left column to the right are *q* = 2.0 to *q* = 4.0 respectively. The standard error is adjusted using Newey-West method.

To fully present the RIA-ACD(1,1) results for all the time series, we present *α*_*q*,1_ s and *β*_*q*,1_ s in Fig. 6. The above findings from Table 2 are consistent with the trend shown in Fig. 6. Generally, when *q* = 2, most estimations are significantly greater than 0 whereas the percentage of this significance slumps when *q* = 4, which is either an indirect evidence that the recurrence interval analysis with the RIA-ACD model somewhat fails to capture the very extreme air pollution occurrences or an indicator that the recurrence intervals of very extreme air pollution occurrence seem untraceable. Technically speaking, the paucity of the recurrence interval series at very extreme level makes it difficult to evaluate and predict. Another notable finding is that *β*_*q*,1_ outweighs *α*_*q*,1_ on the whole. As mentioned above, this immediately shows that by integrating the past temporal information, the autoregressive property is efficiently archived in the conditional expected recurrence interval *ψ*. Finally, when we focus on the AQI series specifically, it’s easy to find that as the threshold improves to *q* = 4, only station 4, 9 and 11′s *α* s are still significant and the *β* values of stations 1, 2, 6, 8 and 12 are insignificant. For the same City of Beijing, the discordancy in the temporal-spatial correlation structure logged by different monitoring stations provides a conformational evidence that the occurrence of extreme air pollution in the same place may be driven by distinct mechanisms in different small areas^36^.

**Table 2:** Partial estimated results of the RIA-ACD(1,1) model.

	*q* = 2.0		*q* = 2.5		*q* = 3.0		*q* = 3.5		*q* = 4.0	
	Exponential	Weibull	Exponential	Weibull	Exponential	Weibull	Exponential	Weibull	Exponential	Weibull
**Panel A: Recurrence series of PM**_**10**_ **in Station 2**										
*ω*_*q*_	0.10**	0.11***	0.08	0.19***	0.31***	0.22***	0.10	0.51***	0.06*	0.04 ***
*α*_*q*,1_	0.08**	0.06***	0.10**	0.14***	0.26***	0.22***	0.12	0.00	0.17*	0.08
*β*_*q*,1_	0.82***	0.75***	0.83***	0.49***	0.46***	0.35***	0.81***	0.00	0.83***	0.83 ***
*k*		0.57***		0.51***		0.46***		0.40***		0.38 ***
*Q*_*q*_(10)	7.22	10.09	7.49	9.79	5.98	6.04	2.70	3.97	1.65	1.77
	(0.70)	(0.43)	(0.68)	(0.46)	(0.82)	(0.81)	(0.99)	(0.95)	(1.00)	(1.00)
**Panel B: Recurrence series of AQI in Station 4**										
*ω*_*q*_	0.05***	0.04***	0.15**	0.16**	0.81***	0.33***	0.06	0.03***	0.60***	0.41 **
*α*_*q*,1_	0.05***	0.03***	0.27**	0.17*	0.40	0.06	0.10	0.03	0.00	0.01
*β*_*q*,1_	0.90***	0.89***	0.66***	0.43*	0.00	0.00	0.88***	0.87***	0.40***	0.00
*k*		0.56***		0.50***		0.42***		0.37***		0.32 ***
*Q*_*q*_(10)	11.63	13.82	5.38	10.43	11.69	13.23	4.34	3.50	4.61	4.59
	(0.31)	(0.18)	(0.86)	(0.40)	(0.31)	(0.21)	(0.93)	(0.97)	(0.92)	(0.92)
**Panel C: Recurrence series of NO**_**2**_ **in Station 7**										
*ω*_*q*_	0.09**	0.05***	0.18***	0.16**	0.23	0.12***	0.54***	0.18***	0.05***	0.64
*α*_*q*,1_	0.19***	0.08***	0.50***	0.44***	0.62	0.32	0.29	0.22	0.00***	0.00
*β*_*q*,1_	0.75***	0.79***	0.50***	0.28**	0.38	0.43***	0.10***	0.15**	1.00***	0.00
*k*		0.52***		0.43***		0.36***		0.31***		0.26 ***
*Q*_*q*_(10)	0.98	1.00	3.68	4.23	3.01	3.02	1.14	0.73	3.00	3.22
	(1.00)	(1.00)	(0.96)	(0.94)	(0.98)	(0.98)	(1.00)	(1.00)	(0.98)	(0.98)

We estimate the above model under five thresholds from *q* = 2.0 to *q* = 4.0 with 0.5 increments each time. The table reports the estimated results using both exponential and Weibull distributions. We also estimate the Newey-West corrected standard errors and label the significance of estimations using asterisks, with *, **, ***representing the statistical significance at 10%, 5%, and 1% level. The Ljung-Box Q-test for residuals autocorrelation (lags = 10) is reported in the last row of each panel and *p*-value of the Ljung-Box statistic is in the parentheses.

**Table 3:** Partial estimated results of the RIA-SACD(1,1,1) model under the Weibull distribution.

	*q* = 2.0		*q* = 2.5		*q* = 3.0		*q* = 3.5		*q* = 4.0	
	Exponential	Weibull	Exponential	Weibull	Exponential	Weibull	Exponential	Weibull	Exponential	Weibull
**Panel A: Recurrence series of PM**_**10**_ **in Station 2**										
${\omega }_{q}^{(i)}$	0.04	0.12***	0.53***	0.34***	0.06**	0.22***	0.06***	0.03**	0.59***	0.27***
${\alpha }_{q\mathrm{,1}}^{(i)}$	0.04***	0.06***	0.26***	0.21***	0.15*	0.23***	0.04	0.01	0.02	0.02
${\beta }_{q\mathrm{,1}}^{(i)}$	0.89***	0.69***	0.12	0.11	0.82***	0.34***	0.80***	0.85***	0.00	0.00
${\gamma }_{q\mathrm{,1,1}}^{(i)}$	0.02	0.04***	0.13***	0.08***	0.02	0.00	0.14***	0.06***	0.42**	0.53
*k*		0.58***		0.51***		0.46***		0.41***		0.39 ***
${Q}_{q}^{(i)}\mathrm{(10)}$	5.01	5.83	11.90	11.31	2.46	1.20	16.64	18.82	0.43	0.71
	(0.89)	(0.83)	(0.29)	(0.33)	(0.99)	(1.00)	(0.08)	(0.04)	(1.00)	(1.00)
**Panel B: Recurrence series of AQI in Station 4**										
${\omega }_{q}^{(i)}$	0.87***	0.04***	0.51***	0.27***	0.70***	0.30***	0.08	0.36***	0.02**	0.41**
${\alpha }_{q\mathrm{,1}}^{(i)}$	0.08***	0.03***	0.62***	0.27**	0.55	0.09	0.05	0.03	0.00	0.01
${\beta }_{q\mathrm{,1}}^{(i)}$	0.00	0.88***	0.05	0.04	0.00	0.00	0.88***	0.00	0.94***	0.00
${\gamma }_{q\mathrm{,1,1}}^{(i)}$	0.09	0.00	0.17***	0.10***	0.10**	0.04	0.01	0.01	0.06	0.01
*k*		0.56***		0.50***		0.42***		0.36***		0.32***
${Q}_{q}^{(i)}\mathrm{(10)}$	55.92	11.91	15.18	15.26	8.52	8.26	2.57	4.13	1.62	3.55
	(0.00)	(0.29)	(0.13)	(0.12)	(0.58)	(0.60)	(0.99)	(0.94)	(1.00)	(0.97)
**Panel C: Recurrence series of NO**_**2**_ **in Station 7**										
${\omega }_{q}^{(i)}$	0.12*	0.07**	0.51***	0.15***	0.19	0.11***	0.41	0.09	0.02	0.29***
${\alpha }_{q\mathrm{,1}}^{(i)}$	0.17***	0.07***	0.12***	0.40***	0.65**	0.38	0.28	0.19	0.00	0.00
${\beta }_{q\mathrm{,1}}^{(i)}$	0.70***	0.75***	0.00	0.32***	0.35*	0.33***	0.29***	0.41*	1.00	0.00
${\gamma }_{q\mathrm{,1,1}}^{(i)}$	0.02	0.01	0.59***	0.00	0.15	0.12	0.05	0.03	0.02	0.29***
*k*		0.52***		0.43***		0.37***		0.31***		0.27***
${Q}_{q}^{(i)}\mathrm{(10)}$	0.97	0.95	2.90	4.08	2.80	2.77	2.25	0.53	2.99	3.34
	(1.00)	(1.00)	(0.98)	(0.94)	(0.99)	(0.99)	(0.99)	(1.00)	(0.98)	(0.97)

We estimate the above model under five thresholds from *q* = 2.0 to *q* = 4.0 with 0.5 increments each time. The table reports the estimated results using both exponential and Weibull distributions. We also estimate the Newey-West corrected standard errors and label the significance of estimations using asterisks, with *, **, ***representing the statistical significance at 10%, 5%, and 1% level. The Ljung-Box Q-test for residuals autocorrelation (lags = 10) is reported in the last row of each panel and *p*-value of the Ljung-Box statistic is in the parentheses.

**Figure 7:**
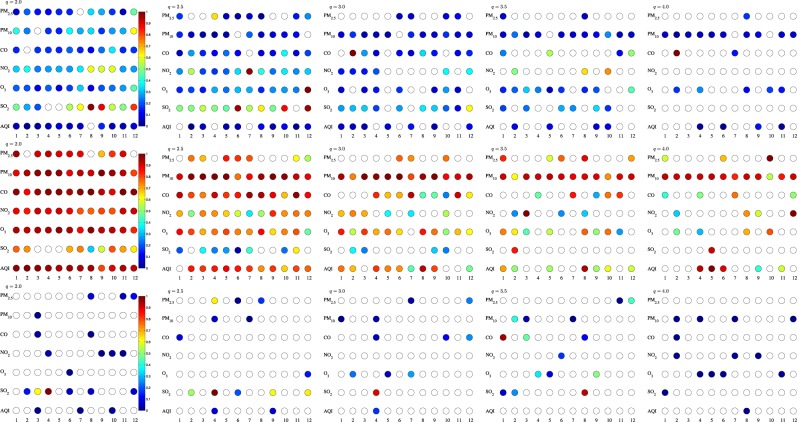
${\alpha }_{q\mathrm{,1}}^{(i)}$, ${\beta }_{q\mathrm{,1}}^{(i)}$ and ${\gamma }_{q\mathrm{,1,1}}^{(i)}$ of each time series estimated from the RIA-SACD(1,1,1) model. The magnitudes of the parameters are represented by different colors filled in each circle, and the circle with no color filled in signifies that the estimated parameter is not significant at 95% level. The top row is the ${\alpha }_{q\mathrm{,1}}^{(i)}$, the middle row is the ${\beta }_{q\mathrm{,1}}^{(i)}$ and the bottom row is ${\gamma }_{q\mathrm{,1,1}}^{(i)}$. Presented from the left column to the right are *q* = 2.0 to *q* = 4.0 respectively. The standard error is adjusted using Newey-West method.

Table 3 incorporates the spatial term into the RIA-ACD model on the basis of Table 2. As mentioned before, the chief reason to adopt this model is to check whether integrating spatial information is helpful in evaluating the extreme air pollution periods. Compared with the results in Table 2, the values of *α* and *β* are generally close to the model without spatial term due to the orthogonal relationship between temporal dimension and spatial dimension. But when it comes to the *γ*’s in Fig. 7, it’s quite obvious that the spatial correlation only exists in 20% of all the selected time series. Moreover, this spatial significance varies with the threshold *q* while the overall percentage of significant spatial terms is close. The difficulty in capturing high extreme pollution level from temporal side has been construed before and the same is also true with *q* from the spatial side. Anyway, though the RIA-SACD model has merely made limited improvements, it still can explain part of the spatial correlations.

### Out-of-sample test

In this section, we conduct a sliding window scheme of out-of-sample test to explore whether the RIA-ACD(1,1,1) model is valid in predicting the recurrence intervals and to what extent the predictive power can be improved using the RIA-SACD(1,1,1) model.

The expected next recurrence duration at threshold *q* using the RIA-SACD(1,1,1) model is given by

3 $$E[{d}_{q,s+1}^{(i)}|{I}_{s}]={\hat{\psi }}_{q,s+1}^{(i)}={\hat{w}}_{q}^{(i)}+{\hat{\alpha }}_{q\mathrm{,1}}\,{\psi }_{q,s}^{(i)}+{\hat{\beta }}_{q\mathrm{,1}}\,{d}_{q,s}^{(i)}+{\hat{\gamma }}_{q,j\mathrm{,1}}^{(i)}\,{d}_{q,s}^{(ij)}$$

For each recurrence interval series ${d}_{q,s}^{(i)}$ with *s* = 1, 2, …, *N*, we choose *s* = 1, 2, …, *N* − 30 as the in-sample estimation, and recursively estimate the next recurrence duration ${d}_{q,s+1}^{(i)}$. By comparing ${\hat{\psi }}_{q,s+1}^{(i)}$ and ${d}_{q,s+1}^{(i)}$, we report the absolute error (AE)

4 $$A{E}_{q,s+1}^{(i)}=|{\hat{\psi }}_{q,s+1}^{(i)}-{d}_{q,s+1}^{(i)}|$$

and the root-mean-square error (RMSE)

5 $$RMS{E}_{q}^{(i)}=\sqrt{\frac{1}{30}\sum _{s\mathrm{=1}}^{30}{({d}_{q,s+1}^{(i)}{\hat{\psi }}_{q,s+1}^{(i)})}^{2}}\mathrm{.}$$

**Figure 8:**
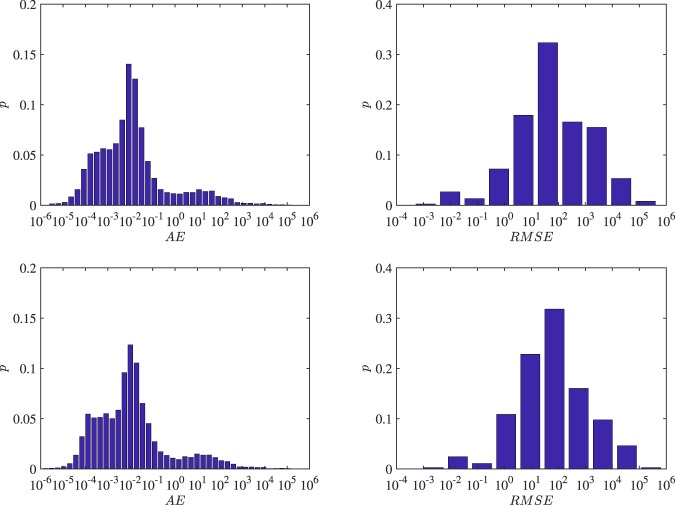
Distributions of *AE* and *RMSE* from the RIA-ACD(1,1) model and the RIA-SACD(1,1,1) model. The top row is the RIA-ACD(1,1) model and the bottom row is the RIA-SACD(1,1,1) model.

**Figure 9:**
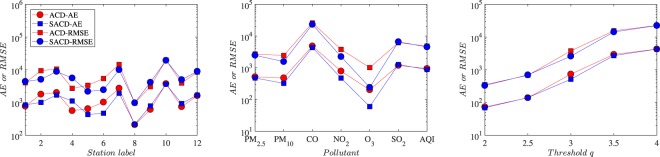
Average *AE* or *RMSE* from the ACD(1,1) model and the SACD(1,1,1) model from three dimensions.

Figure 8 displays the distributions of *AE* and *RMSE* of the out-of-sample test using both the RIA-ACD(1,1) model and the RIA-SACD(1,1,1) model. Together with Table 4, it shows that more than 90% of the predictions deviate from the true recurrence intervals within 3 days under both models, which demonstrates the good predictive power of these models. In terms of the *p* values of *AE* and *RMSE*, incorporating the spatial term does no better than the model without spatial term. However, the average absolute error and root-mean-squared error decrease by 10% if the spatial term is included in the ACD structure. In this sense, the spatial autoregressive conditional model generally performs slightly better than the model considering no spatial interaction. Figure 9 shows the average *AE* or *RMSE* from the ACD(1,1) model and the SACD(1,1,1) model from three dimensions.

**Table 4:** p-values of some AE and RMSE breakpoints and average AEs and RMSEs.

	*p* (*AE* < 1)	*p* (*AE* < 6)	*p* (*AE* < 12)	*p* (*AE* < 24)	*p* (*AE* < 48)	*p* (*AE* < 96)	*Avg* (*AE*)
ACD (1,1)	0.84	0.88	0.89	0.90	0.91	0.93	137
SACD (1,1,1)	0.83	0.88	0.89	0.90	0.91	0.93	123
	*p* (*RMSE* < 1)	*p* (*RMSE* < 6)	*p* (*RMSE* < 12)	*p* (*RMSE* < 24)	*p* (*RMSE* < 48)	*p* (*RMSE* < 96)	*Avg* (*RMSE*)
ACD (1,1)	0.04	0.13	0.18	0.25	0.31	0.44	717
SACD (1,1,1)	0.04	0.13	0.17	0.23	0.30	0.43	642

**Figure 10:**
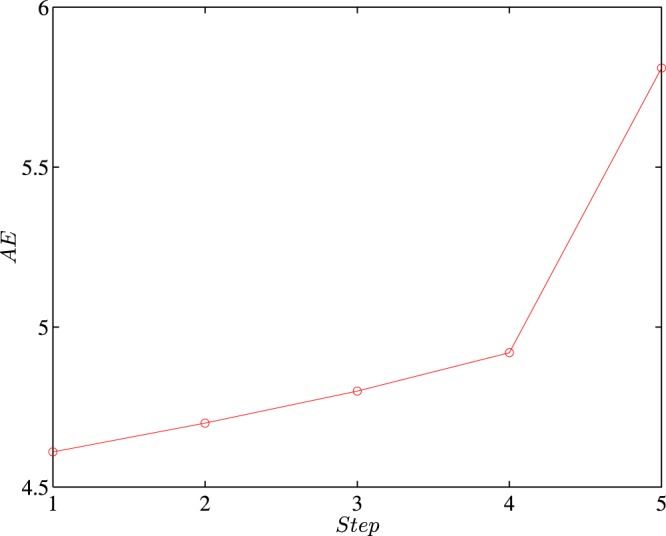
Absolute errors of multi-step out-of-sample test of PM_2.5_ series and station one under the threshold *q* = 2.0. The results are obtained from RIA-SACD model.

To further consolidate the predictive power of the integrated model, our research goes from the one-step forward to multi-step forward. However, as illustrated in Eq. (3) and noted above, a moving window scheme is employed when the out-of-sample prediction is examined. Here, we only present results from the recurrence intervals of PM_2.5_ in station one and under the threshold *q* = 2.0 in Fig. 10 due to limitation of computational consumption. It’s quite straightforward that as we go further steps, the average absolute errors increase, which means that the predictive power will decrease when the most recent information is used to predict the further extreme air pollution event. However, this declining trend of the predictive power is still within our expectation. Specifically, the absolute error that forecasts the most recent approaching extreme air pollution is 30% lower than that forecasts the 5th approaching extreme air pollution from now on. In fact, due to the presence of persistence, one can easily get highly reliable one-step forecasts using a simple persistence model. On the other hand, when making prediction, we intuitively make full use of the past information and make predictions on the nearest future event. Practically speaking, one-step forward is still of great significance in the air pollution prediction.

### More general settings

The above preliminary results have shown that both time dimension and spatial dimension have the ability to capture the structures of recurrence interval series. But the predictability from the temporal dimension is much stronger than the spatial dimension and the predictive power varies with *q*. In this section, the model has been extended to more time lags and more neighbouring stations in order to check the temporal and spatial persistence as well as how the explanatory power is debilitated with lags and distances. On the temporal side, finding out the temporal persistence is vital to identify how extreme air pollution events happen and build up the knowledge of statistics about air pollution. On the spatial side, detecting the relationship between explanatory power and inter-station distances is salutary to understand the spatial interactions of air pollutants.

**Figure 11:**
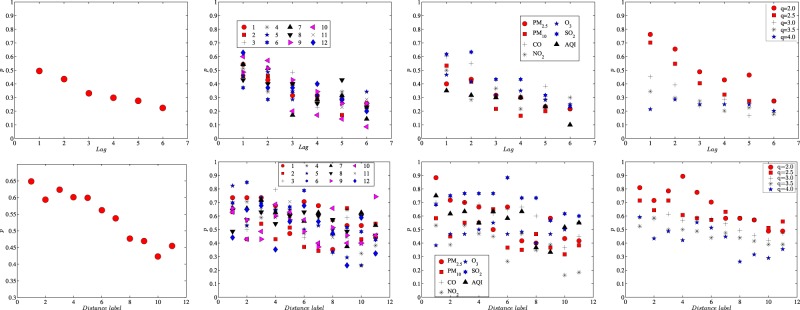
Percentages of significant ${\beta }_{q,v}^{(i)}$ s across lags and significant ${\gamma }_{q,j\mathrm{,1}}^{(i)}$ s across distances based on Eq. (6) and Eq. (7). We first estimate each time series using the above extended models and then calculate the percentage of ${\beta }_{q,u}^{(i)}$ s and ${\gamma }_{q,j\mathrm{,1}}^{(i)}$ s above the 95% significant level in each dimension.

Figure 11(a–d) displays significant *β*s in each lag. These percentages, in other words, measure the Markovian properties of the recurrence intervals in question. Generally speaking, about half of the series show strong autocorrelation in the first lag, and the percentage goes down to only 20% when it comes to the sixth lag. For one thing, the declining persistence accounts for short-term correlations of the recurrence intervals, which supports the exponential distributions of the recurrence intervals. For another, it shows the specifications included in the first several lags are sufficient in the estimation. When classified into three dimensions, the trend in each dimension is consistent with the overall trend in Fig. 11(a). However, some outliers such as those for SO_2_ with *q* = 4.0 at station 5 are found to deviate from this trend. In these outliers, more significant *β*’s are observed in the second lag instead of the first lag,

6 $${\psi }_{q,s}^{(i)}={\omega }_{q}^{(i)}+\sum _{u\mathrm{=1}}^{{L}_{d}}\,{\alpha }_{q,u}^{(i)}{d}_{q,s-u}^{(i)}+\sum _{v=1}^{{L}_{\psi }}\,{\beta }_{q,v}^{(i)}{\psi }_{q,s-v}^{(i)},$$

which suggests that we cannot exclude the possibility that some recurrence interval series possess strong long-term correlations. The factor immediately relevant to the explanatory power is the distance between stations that may be mutually beneficial in explaining and predicting. In the above nearest neighbours’ results, it has been found that the nearest neighbour’s recurrence intervals have predictive power in the station’s recurrence intervals. Here in a more general setting, we go a step further to accommodate all the other stations in the spatial terms of Eq. (7) in recognizing the spatial effects:

7 $${\psi }_{q,s}^{(i)}={\omega }_{q}^{(i)}+{\alpha }_{q\mathrm{,1}}^{(i)}{d}_{q,s-1}^{(i)}+{\beta }_{q\mathrm{,1}}^{(i)}{\psi }_{q,s-1}^{(i)}+\sum _{j\mathrm{=1}}^{11}\,{\gamma }_{q,j\mathrm{,1}}^{(ij)}{d}_{q,s-1}^{(ij)}\mathrm{.}$$

Before performing the following calculation, we first sort out *i*’s spatial neighbours in an ascending order, with *j* = 1 as the nearest station away from *i* while *j* = 11 as the farthest.

After testing all the recorded time series, we display the percentages of ${\gamma }_{q,j\mathrm{,1}}^{(i)}$ s above the 5% significance level in Fig. 11. Generally speaking, it’s cogent that the nearest station possesses the strongest power to explain the recurrence intervals, which has a decreasing trend along with the increasing distance. According to Fig. 11(a), of all the series, around 65% of the nearest neighbours’ spatially reviewed recurrence intervals show a strong relationship with the conditional recurrence intervals and this percentage dwindles to about 40% when it comes to the farthest station. Table 1 reveals that even with the farthest station, they still maintain a high correlation in the original concentration series, which supports 40% of significant spatial interactions. In Fig. 11(b–d), the percentages are presented specifically from three dimensions. Although the overall trend in each dimension is congruent with that in (a), there are still some outliers that deviate from the track. For instance, in the station dimension the most powerful neighbouring stations are mainly the two closest stations, and station 9′s most powerful neighbour is the farthest one instead of the nearest one. In the pollutant dimension, the most powerful station to explain the SO_2_’s trend seems to be neither the nearest nor the farthest one. In the threshold dimension, when *q* = 2, the most powerful station to explain the recurrence intervals may be the fourth closest station. In spite of the data noise and model misspecification, these outliers are partly ascribed to the unexplained spatial interactions. In our setting, one primary assumption is that the strength of spatial interaction is inversely related to the inter-station distance, which is true of the sample population but not necessarily specific individuals. Notwithstanding, it’s quite instrumental for us to get clues on heterogeneous spatial interactions from the above discussions, which in turn profits the choice of a proper *j* in Eq. (12), not simply the nearest neighbour.

## Discussion

Motivated by the methods that model irregular spaced transaction data in the stock market^22,34^, we apply the autoregressive conditional duration model into the recurrence interval analysis of extreme air pollution events. Meanwhile, the spatial interaction and similarity between stations are taken into account and the spatial reviewed recurrence intervals are proposed to check whether the recurrence intervals of neighbouring stations are of high correlations. A simple simulation shows that when the original time series are of high correlations, the generated recurrence interval series are more likely to maintain high correlations, whereby we attempt to add the spatial reviewed term into the autoregressive conditional duration model to fully incorporate the spatial effects. Therefore, this paper copes with two crucial issues. One is to make an attempt to integrate two models which are commonly used in risk assessment and the other is to explore to what extent the spatial interaction can be utilized to predict the value at risk. This paper embarks on the exploration of these two issues from the simplest setting: the RIA-ACD(1,1) model and the RIA-SACD(1,1,1) model. Both the partial results and the general framework show that RIA-ACD is valid in capturing the characteristics of recurrence intervals of different pollutants and the AQI series. However, the predictive power of the proposed models varies in different threshold *q*s. We conclude that as the threshold rises, which means fewer extreme cases, the memory property of the recurrence intervals would become hard to measure due to the lack of enough data. The RIA-ACD and RIA-SACD model will lose some power in capturing the properties. From the spatial side, adding spatial information only increases some recurrence intervals. No matter how the threshold varies, the percentage of the series that have spatial connections is around 20%, which means the use of spatial information to predict the recurrence interval is not pervasively efficient in all pollutants at all stations.

By extending the model to more time lags and farther stations, we find a downward trend of the coefficients’ significance. This temporal and spatial contracting of recurrence intervals is quite consistent with intuition and can partly be explained by some empirical results^37^. This declining significance is also displayed from the station level, the threshold level and the pollutant level. Despite the general trend, some outliers do exist and unveil the spatial transmission and interaction of different pollutants^26^, probably caused by some meteorological conditions^38^.

Although this method provides a statistical direction to identify extreme air pollution occurrence intervals and the results are carefully presented, there are several challenges that require further efforts. The first one is how to identify the best distributions to fit the model. In this paper, we choose exponential and Weibull distribution as a simple case. Although other distributions such as generalized Parato distribution probably provide better fits^33^, the critical issue is how to build in a more adaptive distribution while minimizing the likelihood function. The second challenge as shown in the general setting part is that we don’t integrate temporal lags and distant stations in the model at the same time due to the difficulty of estimation: once more items are involved in this model, the maximization of the likelihood is not convergent any longer, which is also true of the number of parameters. Thirdly, this paper presents the spatial information of recurrence intervals by proposing the spatially reviewed recurrence intervals, which offers a direct means to generate two recurrence interval series of equal length. To completely present the spatial interaction, a more accurate and informative method should be developed. Be that as it may, this paper still provides a promising direction to explore and predict the occurrence of extreme air pollution. In addition, a more general framework that aggregates all the displaced stations and all the effective time lags would be of great significance in improving the predictive power for extreme air pollution events. Since more stations and time lags mean more complexity which might add some fixed or random effects of the model and reduce the predictive power, a Bayesian updating scheme similar to looking one step back with spatial aggregation over multiple stations might help^15^.

## Methods

### Data sets

**Figure 12:**
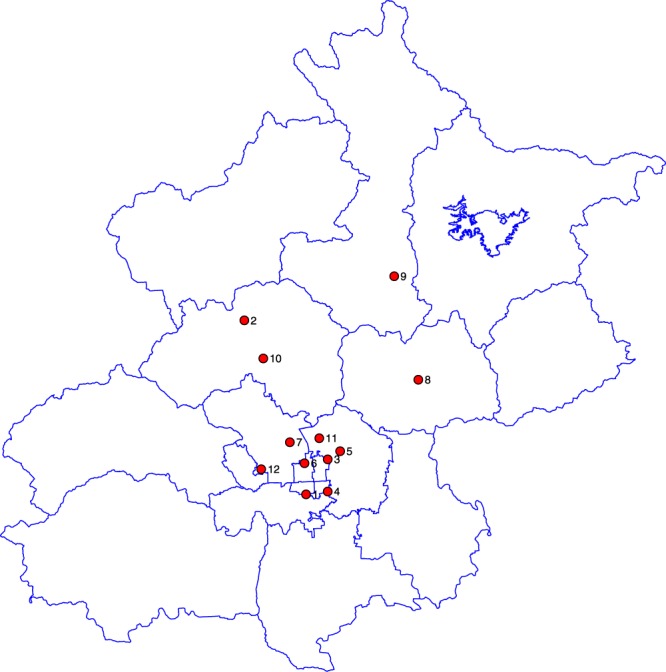
Distributions of the twelve national observation stations in Beijing.

The hourly pollutant data are collected from Shanghai Qingyue Open Environmental Protection Data Center (QOEPDC). We use Beijing as our research object for several reasons. Firstly, the air quality in Beijing, the capital of People’s Republic of China, has always been a focal point of public attention and criticism in recent years. Secondly, air conditions there are more volatile than those in other cities due to a bunch of political reasons. The 12 national air pollutants monitoring stations in Beijing are listed in Table 5, and the geographic distributions of these stations can be found in Fig. 12. We choose the hourly records of PM_2.5_, PM_10_, CO, NO_2_, O_3_, SO_2_ as well as the AQI series in each station as our research samples, spanning from 1 January 2013 to 31 December 2016.

**Table 5:** List of national air pollutants monitoring stations in Beijing.

Label	Code	Lat.	Long.	Label	Code	Lat.	Long.
1	10001A	116.37	39.87	7	10007A	116.32	39.99
2	10002A	116.17	40.29	8	10008A	116.72	40.14
3	10003A	116.43	39.95	9	10009A	116.64	40.39
4	10004A	116.43	39.87	10	10010A	116.23	40.20
5	10005A	116.47	39.97	11	10011A	116.41	40.00
6	10006A	116.36	39.94	12	10012A	116.22	39.93

**Figure 13:**
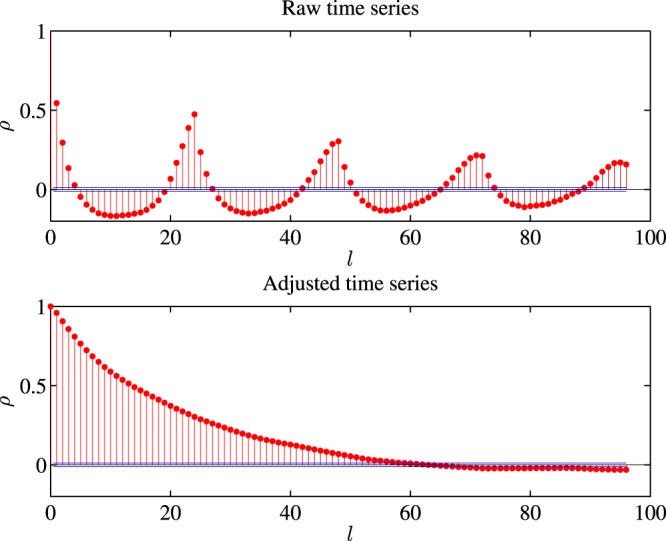
Autocorrelations of two PM_2.5_ time series in station one. (**a**) is the *c* and (**b**) is *x*.

In total, 12 × 7 = 84 time series (AQI included) are collected. The original concentration time series is denoted as *c*(*t*(*d*, *h*)), where *d* is the *d*-th day and *h* is the *h*-th hour of a particular day. In view of the influence of diurnal effect, we remove the intraday effect by dividing the average level at the same hour *h*(*h* = 1, 2, …, 24) in each day

8 $$x(t(d,\,h))=\frac{c(t(d,\,h))}{\frac{1}{{N}_{d}}{\sum }_{d\mathrm{=1}}^{{N}_{d}}\,c(t(d,\,h))},$$

where *N*_*d*_ is the number of days. Through this method, most intraday patterns are removed, and as shown in Fig. 13, the cyclic autocorrelation pattern is transformed into a gradual downward trend. Moreover, this method makes the mean of the series generally close to 1.

### RIA-ACD model and RIA-SACD model

In this section, we first introduce the autoregressive conditional durations (ACD) model which is widely used in analyzing the irregular event durations and then extend the ACD model to a spatiotemporal autoregressive conditional (SACD) model by adding spatial terms in the conditional duration structure.

The ACD model was first proposed from the generalized autoregressive conditional heteroskedasticity (GARCH) model^34^. In the ACD model, the *s*th recurrence interval or duration *d*_*q*,*s*_ is postulated to follow

9 $${d}_{q,s}={\psi }_{q,s}{\varepsilon }_{q,s},$$

where {*ε*_*q*,*s*_} is a sequence of independent and identically distributed random variables and *ψ*_*q*,*s*_ is the expected recurrence interval when all the information set *I*_*q*,*s*−1_ is known. Similar to the GARCH model, the expected recurrence interval is modelled as an ARMA process^34^,

10 $${\psi }_{q,s}={\omega }_{q}+\sum _{u=1}^{{L}_{d}}\,{\alpha }_{q,u}{d}_{q,s-u}+\sum _{v=1}^{{L}_{\psi }}\,{\beta }_{q,v}{\psi }_{q,s-v}.$$

Given that all recurrence intervals are positive, all the coefficients in Eq. (10) must be positive. The logarithmic ACD model has been proposed to avoid this restriction^39–41^. Hautsch also proposed the Box-Cox transformation of Eq. (10) to make the model more adaptive^42^. Since *E*[*ε*_*q*,*s*_] = 1 such that *E*[*d*_*q*,*s*_|*I*_*q*,*s*−1_] = *E*[*ψ*_*q*,*s*_*ε*_*q*,*s*_|*I*_*q*,*s*−1_] = *ψ*_*q*,*s*_, {*ε*_*q*,*s*_} has a positive support and unity expected value. Different distribution assumptions of {*ε*_*q*,*s*_} result in different ACD models^34^.

Based on the ACD model, we propose the spatial ACD model by adding spatial terms in Eq. (10) as follows

11 $${d}_{q,s}^{(i)}={\psi }_{q,s}^{(i)}{\varepsilon }_{q,s}^{(i)}$$

with

12 $${\psi }_{q,s}^{(i)}={\omega }_{q}^{(i)}+\sum _{u=1}^{{L}_{d}}\,{\alpha }_{q,u}^{(i)}{d}_{q,s-u}^{(i)}+\sum _{v=1}^{{L}_{\psi }}\,{\beta }_{q,v}^{(i)}{\psi }_{q,s-v}^{(i)}+\sum _{k=1}^{{L}_{j}}\,\sum _{j=1}^{N(i)}\,{\gamma }_{q,j,k}^{(i)}{w}_{ij}{d}_{q,s-k}^{(ij)},$$

where the added terms include spatially reviewed recurrence intervals ${d}_{q,s-k}^{(ij)}$ with *k* lags and *w*_*ij*_ is the adjacent matrix or deterrence function which is inversely related to the distance^43^.

For simplicity’s sake, we start with the nearest neighbour of location *i*, *NN*(*i*), with one lag behind. So Eq. (12) reduces to

13 $${\psi }_{q,s}^{(i)}={\omega }_{q}^{(i)}+{\alpha }_{q,1}^{(i)}{d}_{q,s-1}^{(i)}+{\beta }_{q,1}^{(i)}{\psi }_{q,s-1}^{(i)}+{\gamma }_{q,1,1}^{(i)}{d}_{q,s-1}^{(NN(i))}$$

In this setting, the adjacent matrix *w*_*ij*_ = 1 if *j* is the nearest neighbour of *i* and *w*_*ij*_ = 0 otherwise. In this paper, the classic ACD model is integrated with the recurrence interval analysis, referred to as the RIA-ACD model to highlight different thresholds. Moreover, Eq. (13) is the case we mainly focus on since this specification is numerically feasible to solve and the properties can be easy to extend. This simplified spatial autoregressive conditional duration model is denoted as the RIA-SACD(1,1,1) model and the model without the spatial terms is hence categorized as the RIA-ACD(1,1) model. The RIA-SACD(1,1,1) model will nest the RIA-ACD(1,1) model if no spatial interaction occurs. However, if two stations are not sufficiently close, the story will be very different. The duration series of the two stations will be “correlated”. The specification also considers the mutual impact on the extreme event occurrence intervals between two locations.

From Eq. (13), we have

14 $$\{\begin{array}{rcl}{\psi }_{q,s}^{(i)} & = & {\omega }_{q}^{(i)}+{\alpha }_{q,1}^{(i)}{d}_{q,s-1}^{(i)}+{\beta }_{q,1}^{(i)}{\psi }_{q,s-1}^{(i)}+{\gamma }_{q,1,1}^{(i)}{d}_{q,s-1}^{(ij)}\\ {\psi }_{q,s}^{(j)} & = & {\omega }_{q}^{(j)}+{\alpha }_{q,1}^{(j)}{d}_{q,s-1}^{(j)}+{\beta }_{q,1}^{(j)}{\psi }_{q,s-1}^{(j)}+{\gamma }_{q,1,1}^{(j)}{d}_{q,s-1}^{(ji)}\end{array}$$

Moreover, $E[{d}_{q,s}^{(i)}]={\psi }_{q,s}^{(i)}=E[{\psi }_{q,s}^{(i)}]$ and $E[{d}_{q,s}^{(j)}]={\psi }_{q,s}^{(j)}=E[{\psi }_{q,s}^{(j)}]$. We obtain that, if two observations *i* and *j* are mutually nearest neighbours (they are close to each other and far away from other stations) and both recurrence interval series are under weak stationary conditions, the expected recurrence interval for station *i* is

15 $$E[{d}_{q}^{(i)}|(j)]=\frac{{\omega }_{q}^{(j)}{\gamma }_{q\mathrm{,1,1}}^{(i)}+{\omega }_{q}^{(i)}(1-{\alpha }_{q\mathrm{,1}}^{(j)}-{\beta }_{q\mathrm{,1}}^{(j)})}{(1-{\alpha }_{q\mathrm{,1}}^{(i)}-{\beta }_{q\mathrm{,1}}^{(i)})(1-{\alpha }_{q\mathrm{,1}}^{(j)}-{\beta }_{q\mathrm{,1}}^{(j)})-{\gamma }_{q\mathrm{,1,1}}^{(i)}{\gamma }_{q\mathrm{,1,1}}^{(j)}}$$

It follows that, if ${\gamma }_{q,1,1}^{(i)}=0$,

16 $$E[{d}_{q}^{(i)}|(j)]=\frac{{\omega }_{q}^{(i)}}{1-{\alpha }_{q\mathrm{,1}}^{(i)}-{\beta }_{q\mathrm{,1}}^{(i)}}\triangleq {\mu }_{i},$$

and if $${\gamma }_{q\mathrm{,1,1}}^{(j)}$$ = 0,

17 $$E[{d}_{q}^{(i)}|(j)]=\tfrac{{\omega }_{q}^{(i)}}{1-{\alpha }_{q,1}^{(i)}-{\beta }_{q,1}^{(i)}}+\tfrac{{\omega }_{q}^{(j)}}{1-{\alpha }_{q,1}^{(j)}-{\beta }_{q,1}^{(j)}}\tfrac{{\gamma }_{q,1,1}^{(i)}}{1-{\alpha }_{q,1}^{(i)}-{\beta }_{q,1}^{(i)}}={\mu }_{i}+{\mu }_{j}\tfrac{{\gamma }_{q,1,1}^{(i)}}{1-{\alpha }_{q,1}^{(i)}-{\beta }_{q,1}^{(i)}}.$$

In the first case, location *j*’s recurrence intervals of extreme events have no impact on *i*’s conditional intervals. Under weak stationary conditions, the expected intervals will reduce to classic ACD expectations. However, in the second case, *i* doesn’t influence *j*, but the other way around (in fact, it can be assumed that directed wind from *j* to *i* always exists). Hence, the first term is a non-spatial term and the second is the spatial interaction triggered intervals.

The next model specification is the distribution of the innovation sequence {*ε*_*q*,*s*_}. With a decreasing trend of the recurrence intervals’ histogram, we resort to the exponential distribution

18 $$f(\varepsilon )={e}^{-\varepsilon }$$

and the Weibull distribution

19 $$f(\varepsilon )=\frac{k}{\lambda }{(\frac{\varepsilon }{\lambda })}^{k-1}{e}^{-{(\varepsilon /\lambda )}^{k}}.$$

In other words, small intervals are more likely than large intervals whatever the threshold *q* is. It should be pointed out that in order to make the Weibull distribution take on this trend, we restrict Weibull’s shape parameter *k* in (0, 1).

Since $E[{\varepsilon }_{q,s}^{(i)}]=1$, we obtain that

20 $$\lambda =\frac{1}{{\rm{\Gamma }}(1+1/k)}.$$

Therefore, the Weibull distribution becomes

21 $$f(\varepsilon )=k{\rm{\Gamma }}(1+1/k){[\varepsilon {\rm{\Gamma }}(1+1/k)]}^{k-1}\,\exp [-{(\varepsilon {\rm{\Gamma }}(1+1/k))}^{k}].$$

#### Maximum likelihood estimation

With the simplest SACD model as an illustrative example, suppose ${D}_{q}^{(i)}=\{{d}_{q,1}^{(i)},{d}_{q,2}^{(i)},\cdots ,{d}_{q,N}^{(i)}\}$ are the realizations of an SACD model. For the parameter set ${\theta }_{q}^{(i)}=({\omega }_{q}^{(i)},{\alpha }_{q,1}^{(i)},{\beta }_{q,1}^{(i)},{\gamma }_{q,1,1}^{(i)})$, the likelihood function of these recurrence interval realizations is

22 $$f({D}_{q}^{(i)}|{\theta }_{q}^{(i)})=f({d}_{q,1}^{(i)}|{\theta }_{q}^{(i)})\prod _{s=2}^{N}f({d}_{q,s}^{(i)}|{d}_{q,s-1}^{(i)},{\theta }_{q}^{(i)}).$$

For the exponential distribution, we have

23 $$f({d}_{q,s}^{(i)}|{d}_{q,s-1}^{(i)},{\theta }_{q}^{(i)})=\frac{1}{{\psi }_{q,s}^{(i)}}\,\exp \,[-\frac{{d}_{q,s}^{(i)}}{{\psi }_{q,s}^{(i)}}].$$

For the Weibull distribution, similarly, we have

24 $$f({d}_{q,s}^{(i)}|\,{d}_{q,s-1}^{(i)},\,{\theta }_{q}^{(i)})=\frac{1}{{\psi }_{q,s}^{(i)}}k{\rm{\Gamma }}(1+1/k){[-\frac{{d}_{q,s-1}^{(i)}}{{\psi }_{q,s}^{(i)}}{\rm{\Gamma }}(1+1/k)]}^{k-1}\,\exp \,[-{(-\frac{{d}_{q,s-1}^{(i)}}{{\psi }_{q,s}^{(i)}}{\rm{\Gamma }}(1+1/k))}^{k}]$$

The conditional log likelihood function of the data becomes

25 $$L({\theta }_{q}^{(i)}|{D}_{q}^{(i)})=-\sum _{s=2}^{N}\,\mathrm{log}\,[\,f({d}_{q,s}^{(i)}|{d}_{q,s-1}^{(i)},\,{\theta }_{q}^{(i)})].$$

The estimate of ${\hat{\theta }}_{q}^{(i)}$ is obtained by maximizing $L({\theta }_{q}^{(i)}|{D}_{q}^{(i)})$ analytically or numerically.

The standard errors of the estimates are obtained through the Fisher information matrix. In view of the effect of serial correlation and heteroskadacity, we adopt the Newey-West robust standard errors by correcting the residuals with the weight of 1 − *s*/*N*^44^.

#### Model diagnosis

Similar to the classic ACD model, we use the Ljung-Box Q as a test statistic for the model^45^:

26 $${Q}_{q}^{(i)}(L)=N(N+2)\,\sum _{k=1}^{L}\,\frac{{\rho }_{{\varepsilon }_{q}^{(i)}}^{2}(k)}{N-k}$$

where ${\rho }_{{\varepsilon }_{q}^{(i)}}(k)$ is the *k*-th lag autocorrelation of ${\varepsilon }_{q}^{(i)}$. If the fitted model is adequate, the standardized innovation ${\varepsilon }_{q}^{(i)}$ should take on an i.i.d sequence of random variables with the assumed distribution. Therefore, there will be no serial correlations detected from the innovations and ${Q}_{q}^{(i)}(L)$ is insignificant. Although some drawbacks have been pinpointed and other statistics are proposed^46^, the Q-test is still the most popular one to check the autocorrelations of the residuals.

## Data Availability

The data are owned by the Shanghai Qingyue Open Environmental Protection Data Center (https://data.epmap.org/). The center provides two options for accessing the data. Interested readers can browse the web site or send an email to support@epmap.org.cn for detailed information.
